# Dorsal penile nerve block for rigid cystoscopy in men: study protocol for a randomized controlled trial

**DOI:** 10.1186/s13063-016-1281-9

**Published:** 2016-03-18

**Authors:** Yan Qiu, An-min Hu, Jin Liu, Gui-zhi Du

**Affiliations:** Department of Anesthesiology, West China Hospital of Sichuan University, #37, Guoxue Ave., Chengdu, Sichuan 610041 China

**Keywords:** Analgesia, Dorsal penile nerve block, Rigid cystoscopy, Men

## Abstract

**Background:**

Pain is common in men undergoing rigid cystoscopy. Even with the application of a lubricant containing 2 % lidocaine, about 76 % of men suffer from mild to severe pain when undergoing rigid cystoscopy. The most painful part of the procedure for men is when the cystoscope passes through the membranous urethra. Song et al. (Neurourol Urodyn 29:592–5, 2010) did autopsies on males and found that the dorsal nerve of the penis (DNP), the terminal branch of the pudendal nerve, innervates the membranous urethra in 53.3 % of specimens. In addition, the urethral mucosa has branches of innervated DNP. Dorsal penile nerve block (DPNB) is usually used for circumcision in children, and it has been shown to provide effective analgesia for penile surgeries. In this study, we hypothesized that DPNB could reduce the overall pain level in men during rigid cystoscopy.

**Methods/design:**

The trial is a prospective, randomized, double-blind, placebo-controlled, single-center trial to evaluate the effectiveness and safety of DPNB in analgesia for men undergoing rigid cystoscopy. Participants will be enrolled and randomly allocated into one of three groups according to the different analgesia regimens: 1) tetracaine gel group (DPNB with saline), 2) DPNB group (DPNB with ropivacaine plus plain lubricant), 3) combination group (DPNB with ropivacaine plus tetracaine gel). The primary outcome of this study is the visual analog scale (VAS, 0–10) for pain at cystoscopic inspection of the external sphincter. VAS scores evaluated at other time points serve as secondary outcomes. Vital signs are secondary outcomes that address the discomfort and pain during the procedure. Furthermore, the incidence of adverse events as secondary outcomes will also be recorded for evaluation of the safety of DPNB in rigid cystoscopy. Clinical assessments will be evaluated prior to DPNB, at administration of the lubricant gel, at cystoscopic inspection of the penile and bulbar urethra, external sphincter, prostate, and bladder, as well as at withdrawal of the cystoscope.

**Discussion:**

This research will determine the effectiveness and safety of DPNB in men undergoing rigid cystoscopy. The results of this trial may have important implications for exploring the role of DPNB in analgesia for cystoscopy in men.

**Trial registration:**

ClinicalTrials.gov identifier NCT02502487 (6 Jul 2015).

## Background

Cystoscopy is a kind of invasive manipulation often used by urologists for diagnosis and treatment of bladder cancer and other lower urinary tract diseases [1, 2]. Flexible cystoscopy cause less discomfort and pain than rigid cystoscopy, but it still cannot replace the rigid cystoscopy technique because of the latter’s lower cost and better optical area, and the fact that it is easy to orient [3].

Pain and discomfort is common in men undergoing rigid cystoscopy. Under general anesthesia, it was reported that 30 % of patients complain of “feeling unwell” within 1 week after rigid cystoscopy [4]. In outpatient settings, even with application of a lubricant containing 2 % lidocaine, about 76 % of men suffer from mild to severe pain when undergoing rigid cystoscopy, and approximately 27 % of patients could still feel mild to moderate pain 7 days after the procedure [5].

Since the urethra is longer and narrower in men, cystoscopy generally tends to be more painful for them [6]. The most painful part of the procedure for men is when the cystoscope passes through the membranous urethra [7]. Song et al. [8] performed autopsies on males and found that the dorsal nerve of the penis (DNP), the terminal branch of the pudendal nerve, innervates the membranous urethra in 53.3 % of specimens. In addition, the urethral mucosa is innervated by branches of DNP [9].

Currently, dorsal penile nerve block (DPNB) is usually used for circumcision in children, and it has been shown to provide effective analgesia for penile surgeries since it was first reported by Kirya and Werthmann in 1978 [10]. To date, whether DPNB could be applied in cystoscopy for analgesia has not been addressed. Since some men have DNP branches innervating the membranous urethra and urethra mucosa, we hypothesized that DPNB could overall reduce the pain level in men during rigid cystoscopy.

## Methods/design

### Trial design

**Fig. 1:**
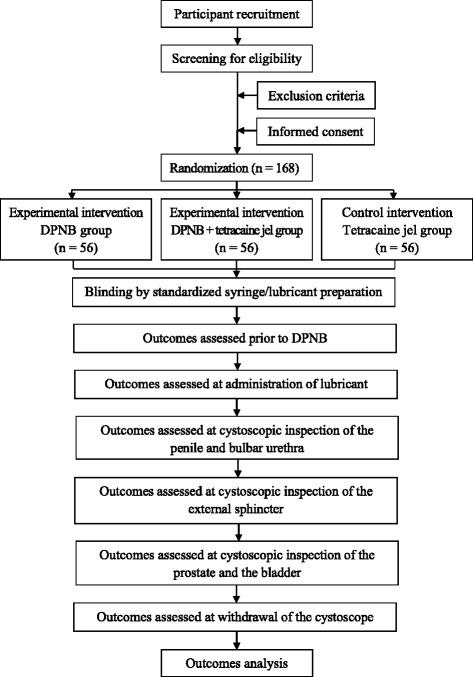
Trial scheme. DPNB, dorsal penile nerve block

The trial is a randomized, double-blind, placebo-controlled, single-center trial to evaluate the effectiveness and safety of DPNB for analgesia in men undergoing rigid cystoscopy. Participants will be randomized to one of three study groups according to different analgesia regimens: 1) tetracaine gel group (DPNB with saline), 2) DPNB group (DPNB with ropivacaine plus plain lubricant), 3) combination group (DPNB with ropivacaine plus tetracaine gel). The trial scheme is illustrated in Fig. 1.

### Ethics

This study was approved by the Clinical Trials and Biomedical Ethics Committee of West China Hospital of Sichuan University (Ref: 20150611) and was registered with the US National Institutes of Health Clinical Trials registry (NCT02502487). This trial will be performed according to the principles of the Declaration of Helsinki (Edinburgh 2000 version). Written informed consent will be obtained from each participant before enrollment.

### Randomization

A set of random numbers for the allocation sequence will be generated using a computerized SPSS software package (version 18; SPSS Inc., Chicago, IL, USA). Sealed, opaque assignment envelopes will be used for allocation concealment. The included participants will be randomly enrolled by sealed envelope and assigned to one of three groups (tetracaine gel, DPNB, or combination group) in a ratio of 1:1:1.

### Blinding

A nurse, who will open the sealed envelopes, will prepare the syringe containing either 0.3 ml/kg of 0.33 % ropivacaine (AstraZeneca Pharmaceutical, Inc., London, UK) or 0.3 ml/kg of saline for DPNB, and 10 ml of 1 % tetracaine gel (Xi’an Lijun Pharmaceutical Co., Ltd., Xi’an, China) or 10 ml of liquid glycerine (YunJia Medical Technology Co., Ltd., Harbin, China) for instillation. The participants, attending anesthesiologist and urologist, as well as the researchers will be not aware of the randomization. The allocation concealment will not be exposed until the final data analysis report is completed.

### Participants

#### Study population

Male patients undergoing rigid cystoscopy aged 20–75 years will be recruited from West China Hospital of Sichuan University. The trial will be ongoing from August 2015 to March 2016.

### Inclusion criteria

- American Society of Anesthesiologists (ASA) Physical Status I–II
- Without history of urethral or prostatic surgery
- Without respiration or circulation disorders
- Without chronic pain

### Exclusion criteria

- Allergy to local anesthetics
- Coagulation disorder or usage of antiplatelet drugs
- Infection at the site of DPNB puncture point
- Severe urethral stenosis

### Interventions

#### DPNB experimental group

For participants randomized to the DPNB group, a DPNB will be performed with a 22-G needle and 0.3 ml/kg of 0.33 % ropivacaine. After skin preparation and palpation of the arch of the lower border of the symphysis pubis, the base of the penis is gently pulled down and the needle is inserted on either side of the midline just distal to the inferior ramus of the pubic bone. The needle is advanced slowly, towards the center of the penile shaft, until loss of resistance is felt as it penetrates Scarpa’s fascia, where the local anesthetic is deposited equally on each side. All blocks will be performed by only one experienced attending anesthesiologist, who is good at the block procedure. Five minutes after DPNB, 10 ml of liquid glycerine will be instilled in the DPNB group at least 3 min before rigid cystoscopy.

#### Experimental group using combination of DPNB and tetracaine gel

In the DPNB and tetracaine gel combination group, a DPNB with 0.33 % ropivacaine (0.3 ml/kg) is performed as in the DPNB group. Five minutes after the DPNB, tetracaine gel is instilled, with a minimum dwell time of 3 min, before rigid cystoscopy.

#### Tetracaine gel control group

For men in the tetracaine gel group, DPNB is performed with saline. Five minutes after DPNB, tetracaine gel is instilled, with a minimum dwell time of 3 min, before rigid cystoscopy.

### Outcomes

#### Primary outcome

The primary outcome of this study is intensity of pain measured by a visual analog scale (VAS) at cystoscopic inspection of the external sphincter. VAS is an internationally recognized pain scale with 11 points, ranging from 0 (no pain) to 10 points (maximal pain). Pain can be rated as the following categories: no pain (0 points), mild pain (1–3 points), moderate pain (4–6 points), and severe pain (7–10 points). Participants will be well educated regarding the VAS once enrolled and asked to rate their pain level during the study.

#### Secondary outcomes

VAS scores, assessed prior to DPNB (baseline), at administration of lubricant, at cystoscopic inspection of the penile and bulbar urethra, the prostate and the bladder, and at withdrawal of the cystoscope, serve as secondary outcomes. Additionally, vital signs (heart rate, blood pressure, breath rate, and pulse oxygen saturation) are secondary outcomes that consider the discomfort and pain during the procedure and will be evaluated at the same time points as the VAS evaluation. Furthermore, the incidence of adverse events (penile hematoma, penile erection, autonomic movement and local anesthetic toxicosis) as secondary outcomes will also be recorded from the beginning of DPNB to the end of cystoscopy for evaluation of the safety of DPNB in rigid cystoscopy.

### Data collection

Each participant’s characteristics such as age and body weight as well as ASA grade, durations of DPNB and rigid cystoscopy, VAS scores, vital signs, and the incidence of adverse events during rigid cystoscopy will be recorded in a designed data form by an investigator.

### Sample size

The sample size calculation was based on the primary outcome, the intensity of pain measured on the VAS at cystoscopic inspection of the external sphincter. There are no data regarding VAS score for men when the rigid cystoscope passes through the membranous urethra. We therefore conducted a pilot study to determine the sample size prospectively. Based on our pilot data and literature relevant to pain during male rigid cystoscopy [11], assuming a standard deviation (SD) of 2.6, an α of 0.05, and a power of 90 %, 56 patients in each group are required to demonstrate a clinically relevant difference of 1.6 units in VAS score at cystoscopic inspection of the external sphincter.

### Statistical analysis

Statistical analysis will be performed with the SPSS (version 18; SPSS Inc., Chicago, IL) and SAS (version 9.3; SAS Inst. Inc., Cary, NC). All statistical tests will be two-sided, and the level of significance will be set at 0.05. Data with a normal distribution will be expressed as mean ± SD and tested by one-way ANOVA with Dunnett’s post hoc test as appropriate. Continuous data without a normal distribution will be expressed as median with interquartile range and analyzed using the MIXED procedure of the SAS statistical program for repeated measures followed by Tukey-Kramer adjustments.

## Discussion

Cystoscopy plays an important role in both diagnosis and treatment in urology. The rigid cystoscope is still applied in many health care centers because of its lower cost and better optical area and because it is easy to orient compared with flexible devices. However, due to the stiffness of the rigid cystoscope sheath, there is discomfort and even pain resulting from the friction between the wall of the urethra or bladder mucosa and the sheath, and patients sometimes cannot finish the cystoscopy, leading to misdiagnosis or missed diagnosis of the disease [1, 2]. Recently, topical intraurethral lidocaine gel, which is used as a local anesthetic, is most commonly used in clinics to ease the pain of the patients, and this gel also could play a role in lubricating the urethra. However, some reports indicated that pain and discomfort is common in men undergoing rigid cystoscopy even with topical administration of local anesthetic or under general anesthesia [4–6]. Thus, an effective analgesia regimen seems to be urgent for men undergoing rigid cystoscopy.

Men suffer more pain than women in cystoscopy. The most painful part of the procedure for men is when the cystoscope passes through the membranous urethra [7]. Since evidence suggests that some men have DNP branches innervating the membranous urethra and urethra mucosa, could DPNB be effective in relieving the pain for men undergoing rigid cystoscopy, especially when the cystoscope passes through the membranous urethra? To address this question, we designed this trial to evaluate the effectiveness and safety of DPNB for analgesia in men undergoing rigid cystoscopy. The results of this trial may have important implications for exploring the role of DPNB in analgesia for cystoscopy in men.

### Trial status

The trial is currently in recruitment phase. Trial completion is expected by March 2016.

## Abbreviations

ASA: American Society of Anesthesiologists
DNP: dorsal nerve of the penis
DPNB: dorsal penile nerve block
SD: standard deviation
VAS: visual analog scale
